# Essays on Various Subjects of Medical Science

**Published:** 1825-01-01

**Authors:** 


*Essays on various Subjects of Medical Science.* By DAVID HOSACK, M.D. F.R.S. &c. &c. Two Volumes, 8vo. New York, 1824.

Of the various papers published in these two volumes, many, we might say most, have already appeared before the public, either in this country or America. Dr. Hosack has been rather remiss sometimes in not indicating with precision, where and when his Essays made their first appearance, and we have not the power of supplying the deficiency. As Dr. Hosack, however, is a man whose facts are entitled to our credence, and whose opinions command our respect, we shall glance over several of the papers contained in this work, and hope that our time will not be deemed by our readers to have been unprofitably spent in the operation.

The first volume is occupied chiefly with subjects of either a local, a temporary, or an elementary nature, which it will not suit our purpose to notice in this article. The second paper in the second volume is entitled "Observations on Fever," and contains many judicious reflections, with some appropriate criticisms on the various doctrines which have been maintained in respect to this disease. He observes that fevers, to this day, constitute the great outlets to human life, and are "almost as fatal as in the time of Sydenham." However minutely we may be acquainted with the symptoms and phenomena of fever, as well as its predisposing and exciting causes, we know very little about its *proximate cause*.

"Whence, then," says Dr. H. "has arisen the discordant, and, we may almost say, the empirical practice, that fills the pages of the best writers on fevers, and that is even to be found in the truly valuable works of Boerhaave, Cullen, Fordyce, Wilson, and others? We answer; it is in a great degree ascribable to the local views of the animal economy to which some of those writers have been limited by their own hypotheses, and which practitioners, relying upon the authority of great names, have hastily adopted." 90.

Boerhaave and Cullen, each run into a dangerous extreme—the first in respect to the humoral, and the second in respect to the nervous pathology. "The still more recent writings of Brown, Beddoes, Darwin, Girtanner, Clutterbuck, Rush, and others, have been too successful in spreading these partial views of the human structure, and consequently limited pathology of the diseases to which it is liable."

"Fever, in the opinion of the writer of these remarks, is a disease of the whole system; it appears no less in all the faculties of the mind than in all the functions of the body; it shows itself in every organ of our frame, and affects every nerve and fibre of the system; the absorbing, the circulating, and excreting systems of vessels, are all affected by it; it shows itself in all the various fluids of the body, as well as in the solids; in a word, it is omnipresent; it has no one pathognomonic symptom, but is constituted by a concourse of symptoms, and these variously combined in the various forms that fever assumes, depending upon the causes whence it proceeds, and the condition of body in which it occurs. If this view of the subject be correct, it will necessarily lead the physician to more extensive principles of practice; it will lead him, at the bed-side of the patient, to pay due regard to the nervous system, the phenomena it exhibits, and the indications thence arising; but at the same time it will lead him to notice the changes which may be induced in the secretions and excretions, and the circulating mass from whence they proceed." 91.

The above is nearly the doctrine which we have maintained in this Journal since its commencement, and the longer we tive, the more we are convinced of the danger as well as error of the partial doctrines which have been so industriously circulated of late years.

Our author properly observes that fever cannot long continue without inducing debility of the vascular as well as other parts of the system, with a corresponding increase of sensibility to impressions. The heart and arteries are then more readily acted on, even by the natural stimuli of the system.

"The heart and vessels are accordingly excited to preternatural frequency, even operated upon by the blood and other fluids of the system in their natural and healthy condition, as we see daily illustrated in the progress of all fevers, and in convalescence from fever: we contend that fever, long continued, not only wastes the power of the solids, rendering them more irritable, but by the derangement in the functions and excretions, perhaps by the action of the blood-vessels themselves upon their contents, and especially by the retention of those materials which should have been thrown out of the system as noxious, which in health are constantly ejected, the circulating fluids become changed and vitiated, and thereby become additional sources of irritation to the heart and arteries, whose susceptibility of impression, as we have just observed, is also morbidly increased. From this view of the more irritable state of the circulating system, and the vitiated condition of the fluids, we infer, that unless by some salutary power inherent in the system itself, or by some means suggested by art, the greater irritability of the whole system, and of the heart and arteries in particular, be diminished, or the morbid changes induced in the fluids they circulate, be counteracted, these causes of fever, mutually operating upon each other, must increase, and fever be continued until the vital principle itself be totally expended.—How far, then, we ask, is the attention of physicians directed to these two cardinal objects, in the treatment of the advanced stage of fevers? how far is their practice calculated either to impart vigour to the system, and thereby to lessen the morbid sensibility of the nervous and moving fibre, or to counteract the septic tendency of the circulating fluids which obtains in most fevers of the continued type?" 94.

Our author is hereby led to condemn that indiscriminate and long-continued use of the debilitating evacuants, usually prescribed at this advanced period of fevers and febrile diseases, in as far as they are calculated to add to that waste of excitement, and that very vitiation of the fluids above referred to. These observations are deserving of attention by the younger practitioners, who are obliged to go by what they are taught, in the first years of their career.

2. *Glossitis.* A melancholy case of this disease is related by Dr. Hosack. A gentleman, Mr. Hamilton of Poughkeepsie, was exposed to great heat in driving his own carriage, and then imprudently threw off his clothes and sat in a stream of cold air. He was soon taken ill, and three days afterwards was found by Dr. Cooper with his tongue swelled, so as to press against the roof of his mouth, and soon afterwards to protrude beyond the teeth. He could not now close his mouth, or articulate, and deglutition was attended with difficulty and distress. Not only the tongue, but the sublingual glands, and the whole neck from the jaw to the clavicles, became very much enlarged. Dr. C. proposed venesectiou, to which Mr. Hamilton would not submit, because he was subject to gout. Purgatives were then administered, and a blister applied. But the symptoms all increased in violence. Dr. Bard was called in, and, on examination, found a suppurating tumour under the tongue. This was opened, and a small quantity of offensive pus discharged. Some abatement of the symptoms now took place, but the relief was transient and deceitful. Drowsiness and coma soon came on, apparently occasioned by congestion of the cerebral vessels.—Delirium soon supervened—his face became of a dark red colour—pulse frequent and full—respiration hurried—discharges from the mouth very offensive. Eight days had now elapsed since the commencement of the disease, but still it was deemed advisable to draw blood from a vein. Fourteen ounces were taken, and a saline cathartic administered. By these he was somewhat relieved; but in the evening, all the symptoms again increased in violence. Next morning, however, appearances were more favourable. The tongue became cleaner, and was much diminished in size, so that he could now bring his teeth together. Still the tumour was hard and unyielding, the breathing was oppressed, and there was some degree of coma. A large blister was applied. In the evening, omnia exacerbata —and a little after midnight he breathed his last.

Dr. Hosack, who was called in some days before the patient's death, augured badly from the first time he saw him, having seen some other instances of a similar nature turn out fatal. The following more recent, was also a more fortunate case.

G. Jerault, a stout man, aged 35, was admitted into the New York Hospital, on the 19th June, 1822, for an attack of irregular fever, which was treated by laxatives and diaphoretics.—This man had an enlargement of the lower jaw and tongue, so as to impede his speech, and which had arisen, it seemed, from a severe salivation induced one or two years before. The disease for which he had taken the mercury had been cured, but the chronic enlargement remained. After recovering from the fever, he again caught cold, and again was seized with febrile symptoms, immediately followed by an extraordinary swelling of the tongue, causing it to protrude more than an inch from the lips, and greatly obstructing respiration. The progress of the enlargement was very rapid—the face was flushed—and the pulse high. He was immediately bled to between 30 and 40 ounces—deep scarifications were made with a scalpel in the tongue—a blister applied to the throat, and antimoniated purges given. These means were almost simultaneous, and they quickly produced relief. By a continuance of them the patient was soon well, as far as the tongue was concerned.

We here see the difference between riches and poverty.—Mr. Hamilton would not submit to those measures which saved the life of his less opulent countryman. In cases of glossitis, local detractions of blood by means of leeches or cupping, should be strictly attended to, as they are of far more consequence than general bleeding.

3. *Phlegmatia Dolens.* We took occasion, in our second number, to differ in sentiment with Dr. Davis respecting the pathology of phlegmatia dolens. In the present volume we have a Commentary on Dr. Davis's paper by the American Professor, from which we shall make only a single extract, shewing the coincidence of opinion between Dr. Hosack and ourselves.

"Such are the facts adduced by Dr. Davis: but while it is acknowledged, that in this communication the author has made large and valuable contributions to the stock of knowledge, collected upon this interesting subject; the question now arises, how far do the facts he has disclosed warrant the inferences he has deduced from them? How far is the conclusion a legitimate one, that because the femoral and iliac veins partake of the derangement affecting almost every part of the limb, that they are necessarily the primary seat and source of the disease; and that the proximate cause, exclusively consists in an inflammation and obstruction of those vessels in particular? With due respect to Dr. Davis, I must be permitted to apply to his reasoning the same observation he has made, with regard to the dissection made by Zinn, and the inferences deduced from it by that author. 'In an analysis of this dissection,' says Dr. Davis, 'it is important to distinguish between the facts that are reported, and the opinion of the writer, as to the order of their relation to each other as cause and effect. In admitting, therefore, the fact of a diminution of diameter in the vein, we are by no means bound by the author's opinion as to the cause.' In like manner I observe, that the inferences deduced by Dr. Davis from the dissection, demonstrating the presence of inflammation in the principal veins of the thigh and pelvis, are altogether gratuitous, and to be received as a matter purely of opinion. And upon a careful examination of the general observations which the Doctor has added, in application of the facts which his dissections have developed, to the phenomena of the disease, I have not been enabled to discover a single argument to show that it commences in the particular vessels thus affected; on the contrary, many circumstances are stated which manifestly lead to an opposite conclusion." 228.

4. *Gout.* Dr. Hosack is led to conclude, *first*, that gout is not an hereditary disease, in the common sense of the term, but only so, as far as fortune, and its attendants, indolence, luxury, and intemperance, are hereditary. In this we do not agree with our intelligent author:—for although it is observed by Hoffman, that—"many have lost their gout with their fortune;" yet it has been observed by many others that this has not been the case; but that the sins of the fathers have been visited on the children to the third and fourth generation.—*Secondly*, he concludes that gout, for the most part, takes place in the sanguine temperament, in the plethoric habit of body, and "is exclusively an inflammatory disease of the whole system, as well as of the part affected."

"3d. That its associates, or vicarious diseases, apoplexy, palsy, angina pectoris, asthma, habitual catarrh, eruptions on the skin, obstructed viscera, and dropsy, arise from the same habit of body, and from the same causes.

"4th. That the deposits of saline or earthy matter, which take place upon the joints in gout and rheumatism, in the kidneys and bladder, occasioning stone and gravel, in the brain of apoplectics, in the arteries of persons advanced in life, in the coronary vessels and valves of the heart, as frequently attendant upon angina pectoris and other diseases of that organ, have the same common origin, and that these extravasations are usually the effects of an overloaded state of the blood-vessels.

"5th. That although the same earthy or saline materials exist in the blood in a state of health, and are constantly passing off in our excretions, as appears from the observations and experiments of Scheele, Wollaston, Brande, Pearson, Egan,* and others, they are in no instances the cause of gout; but when deposited upon the joints in that disease, or upon other parts of the body, that such deposits are the effects of plethora, the parent of both.

"6th. That the predisposing causes of gout in addition to the original vigour of frame, are the excessive use of wine, ardent spirits, animal food, the condiments of the table, and the neglect of the exercise necessary to counteract their effects upon the constitution. While the check of the excretion by the cold of autumn and winter, or the sudden impetus given to the circulation by the returning spring, prove the most usual exciting causes of this disease." 235.

---

* "Transactions of the Irish Academy."

7th. That, as the causes of gout are intemperance and indolence, the best preventives are temperance and exercise.

8th. That the most effectual means of treatment, in the first stage of the gouty paroxysm, consist "in depletion by the lancet, cathartics, and such remedies as operate by restoring the excretions from the surface of the body."

9th. That the part or parts affected should be lightly covered with flannel, during the febrile stage of the paroxysm, for the purpose of soothing irritation, and promoting a perspiration from their surface. Loading the limb with stratum superstratum of flannel, he equally condemns with the application of cold water.*

10th. That after the attack, tonics, bitters, restoratives, &c. are proper.

11th. That the eau médicinale is either useless or injurious.

Our readers are aware that the opinions and practice broached by Dr. Hosack, though not accordant with the sentiments of some living physicians who have written on gout, are nevertheless sanctioned by great names, among which may be reckoned, Sydenham, Huxham, Cullen, Musgrave, Rush, and last not least, the late Dr. Parry of Bath.

5. *Angina Pectoris.* Dr. Hosack is mistaken in supposing that angina pectoris is considered generally by pathologists as depending on ossification of the coronary arteries. It is well known that various changes of structure in the heart—and even disorders of function, will induce the phenomena of the disease. Dr. H. has met with some cases attended with the most alarming symptoms, in which, after the paroxysms were removed by the usual diffusible stimuli, blood-letting and other depletory means were successfully employed, and ultimately effected a cure. From these and other cases which he has seen or read of, he has come to the firm belief that a plethoric condition of the sanguiferous system, both general and local, is to be looked upon as having, if not an exclusive, at least a predominant agency in the production of angina pectoris. This is too limited a view of the subject. The phenomena of the disease, as we remarked before, are the result of various organic as well as functional derangements.

6. *Secale Cornutum.* Dr. Hosack prescribed this remedy

* "Every case," says Dr. Hosack, "which has come within my knowledge, where the practice of immersing the limb in cold water at the invasion of gout has been habitually pursued, has ended in apoplexy or palsy."

in three cases of long protracted labours, wherein all the ordinary resources had been exhausted (the use of instruments excepted) and the patient's strength had become expended. It was given in infusion, and in the usual doses. Its effect was, within fifteen minutes after its exhibition, to excite the uterus to violent action, which did not cease but with the expulsion of the child. The children were still-born in these and in all the other cases which Dr. Hosack has seen of the administration of the ergot. "The ergot has been called, in some of the books, from its effects in hastening labour, the *pulvis ad partum*:—as it regards the *child*, it may, with almost equal truth, be denominated the *pulvis ad mortem*." Dr. Chapman, however, states that it has been administered by himself and others in a hundred instances, without doing harm in any respect. Dr. Hosack apprehends, that, in these cases, the impediment to labour has been so inconsiderable that the child was readily expelled under the influence of the ergot, and where perhaps no necessity existed for its exhibition. Granting that this surmise is correct, it only proves that it is not the ergot which destroys the life of the child, but the difficulties which it meets in its expulsion from the womb.

While Dr. Hosack is inclined to proscribe the ergot altogether, in cases of difficult parturition, preferring the forceps, he conceives that, from the active operation of this medicine on the womb, it promises to be very extensively useful in counteracting many morbid conditions to which that viscus is liable, especially those proceeding from an inactivity in its muscular powers, or lax state of its vessels, as in retention of the placenta—excessive discharges of the lochia—fluor albus—uterine hæmorrhage. An instructive case of this last is here related, and as it is very short, we shall give it in the author's own words:—

"Mrs. L——, a lady about fifty years of age, of a plethoric but relaxed habit of body, ceased to menstruate about four years ago. Since that time she has evinced the ordinary symptoms of fulness of the blood-vessels, that frequently supervene upon the cessation of that function; viz. a sense of distention and pain in the region of the uterus; a fulness of the breasts; oppression of the chest; frequent headache and giddiness. By blood-letting these have been several times removed: but neglecting this remedy when those feelings returned, uterine hemorrhage was the result, followed by great loss of strength. In this debilitated state, in which further depletion was forbidden, I had recourse to the various vegetable and mineral astringents, administered internally and externally, but without effect; cold also was applied to no purpose.—In this lax state of the uterine vessels, I directed ten grains of the ergot to be given in substance three times a day. In a few minutes after the exhibition of each dose, it was followed by the uterine nisus, which immediately stanched the discharge. In a few days, by suitable nourishment, and the continuance of the tonics which she had before employed without the effect of restraining her discharges, she recovered her strength.

"In the course of the last month, she experienced some return of the hemorrhage; but, by a few doses of the ergot, the discharge was as immediately restrained as it had been in the first attack." 300

7. *Death of Mr. Cooke, the Tragedian.* It is well known that this celebrated actor was excessively intemperate in drink. He died in America of dropsy, and apparently enlarged liver. On dissection, the medical attendants were surprised to find the liver not exceeding the usual dimensions. "It was, however, astonishingly hard, and of a much lighter colour than is natural to that organ." It's texture was so dense as to make considerable resistance to the knife. In its internal structure it was so hard and unyielding, that very few traces of its vessels could be found, and the circulation through it had evidently long since ceased to be regularly performed. It exhibited precisely that peculiar tuberculous appearance which was first pointed out by the late Dr. Baillie. The tubercles were not confined to the surface, but extended throughout the greater part of the substance of the liver.

We must now draw our short notice of Dr. Hosack's volumes to a close. A great part of the second volume is occupied with observations on the weather and diseases of New York, which can only be interesting to our transatlantic readers, and they have the original work. We therefore take leave of our able author with every sentiment of respect and esteem.

